# The effect of activation rate on left atrial bipolar voltage in patients with paroxysmal atrial fibrillation

**DOI:** 10.1111/jce.13282

**Published:** 2017-09-19

**Authors:** Steven E Williams, Nick Linton, Louisa O'Neill, James Harrison, John Whitaker, Rahul Mukherjee, Christopher A. Rinaldi, Jaswinder Gill, Steven Niederer, Matthew Wright, Mark O'Neill

**Affiliations:** ^1^ Division of Imaging Sciences and Biomedical Imaging King's College London; ^2^ Cardiovascular Division Guy's and St. Thomas’ NHS Foundation Trust

**Keywords:** atrial arrhythmias, atrial fibrillation, cardiac mapping, catheter ablation, electrogram analysis, three‐dimensional systems

## Abstract

**Introduction:**

Bipolar voltage is used during electroanatomic mapping to define abnormal myocardium, but the effect of activation rate on bipolar voltage is not known. We hypothesized that bipolar voltage may change in response to activation rate. By examining corresponding unipolar signals we sought to determine the mechanisms of such changes.

**Methods and results:**

LA extrastimulus mapping was performed during CS pacing in 10 patients undergoing first time paroxysmal atrial fibrillation ablation. Bipolar and unipolar electrograms were recorded using a PentaRay catheter (4‐4‐4 spacing) and indifferent IVC electrode, respectively. An S1S2 pacing protocol was delivered with extrastimulus coupling interval reducing from 350 to 200 milliseconds. At each recording site (119 ± 37 per LA), bipolar peak‐to‐peak voltage, unipolar peak to peak voltage and activation delay between unipole pairs was measured. Four patterns of bipolar voltage/extrastimulus coupling interval curves were seen: voltage attenuation with plateau voltage >1 mV (48 ± 15%) or <1 mV (22 ± 15%), and voltage unaffected by coupling interval with plateau voltage >1 mV (17 ± 10%) or <1 mV (13 ± 8%). Electrograms showing bipolar voltage attenuation were associated with significantly greater unipolar voltage attenuation at low (25 ± 28 mV/s vs. 9 ± 11 mV/s) and high (23 ± 29 mV/s vs. 6 ± 12 mV/s) plateau voltage sites (P < 0.001). There was a small but significant increase in conduction delay between unipole pairs at sites showing bipolar voltage attenuation (P = 0.026).

**Conclusions:**

Bipolar electrogram voltage is dependent on activation rate at a significant proportion of sites. Changes in unipolar voltage and timing underlie these effects. These observations have important implications for use of voltage mapping to delineate abnormal atrial substrate.

## 1 INTRODUCTION

Left atrial bipolar voltage is a commonly‐used metric to determine the presence of fibrotic atrial myocardium during electrophysiology procedures. The presence of low voltage regions has been associated with arrhythmia recurrence after AF ablation,^1^ persistent forms of AF,^2^, ^3^, ^4^ and has recently been used as a tool to define targets for ablation.^5^, ^6^, ^7^ Despite these associations, the relationship between low voltage, defined by a static voltage threshold, and atrial fibrosis is limited. Indeed, in cardiac MRI studies, atrial enhancement can be identified in both low and high voltage zones,^8^ and the association between CMR indices of fibrosis and voltage is relatively weak.^9^, ^10^ Although voltage mapping has been validated in postablation porcine atrial models,^11^ histological evidence linking low voltage and preablation atrial fibrosis (consequent on or contributing to AF) is currently lacking.

In addition to the presence or absence of fibrosis, low atrial voltage has also been associated with a number of other components of the AF substrate, including high atrial wall stress,^12^ reduced conduction velocity^13^ and increased LA size.^14^ Rhythm during mapping is also linked to alterations in recorded voltages with higher voltages seen during macroreentrant tachycardia than during sinus rhythm,^15^ and lower voltages seen during AF compared to sinus rhythm,^16^ in the same atria. Taken together, these findings suggest that the relationship between voltage and structural atrial change is likely to be complex and may not therefore be well represented by a simple threshold level.

Several pieces of evidence suggest that bipolar voltage may change with activation rate. Firstly, bipolar voltage is lower during pacing at a rate greater than the sinus rate than during sinus rhythm in both AF and non‐AF patients, although the reduction in voltage is greater in AF than non‐AF patients.^17^, ^18^ Second, rapidly activating LA sites during AF (characterized by shorter cycle length and higher dominant frequency) are associated with lower voltage than other sites.^19^ Finally, fractionated electrogram sites that are characterized by low voltage during AF, frequently revert to high bipolar voltage during sinus rhythm.^20^

In this study, we therefore hypothesized that LA bipolar voltage in patients with AF is activation rate dependent, and that changes in bipolar voltage could be revealed by extrastimulus pacing. We sought to define categories of bipolar voltage curves based on the lowest plateau voltage reached at short coupling intervals and the rate of voltage attenuation at long coupling intervals. By recording simultaneous unipolar electrograms, we sought to determine whether extrastimulus coupling interval‐dependent changes in bipolar voltage are explained by changes in local conduction, unipolar voltage or both.

## 2 METHODS

### 2.1 Patient selection and clinical procedures

Ethical approval was granted by the National Research Ethics Service (10/H0802/77) and all participants gave written informed consent for inclusion in the study. The research conformed to the principles described in the Declaration of Helsinki. Patients with ischemic heart disease, cardiac surgery or structural heart disease were excluded. Antiarrhythmic drugs, including calcium channel blockers, were stopped at least five half‐lives before ablation. Amiodarone was stopped at least 6 weeks prior to ablation. All clinical procedures were performed under general anesthesia. Following femoral access and transseptal puncture, two 8.5 French SR0 long sheaths and a PentaRay mapping catheter (Biosense Webster, CA, USA; 1 mm electrode size, 4‐4‐4 mm spacing) were advanced into the LA. Decapole (St Jude Medical, MN, USA) and pentapole (Bard Electrophysiology, MA, USA) catheters were positioned in the coronary sinus and high right atrium, respectively.

### 2.2 Pacing protocol

**Figure 1:**
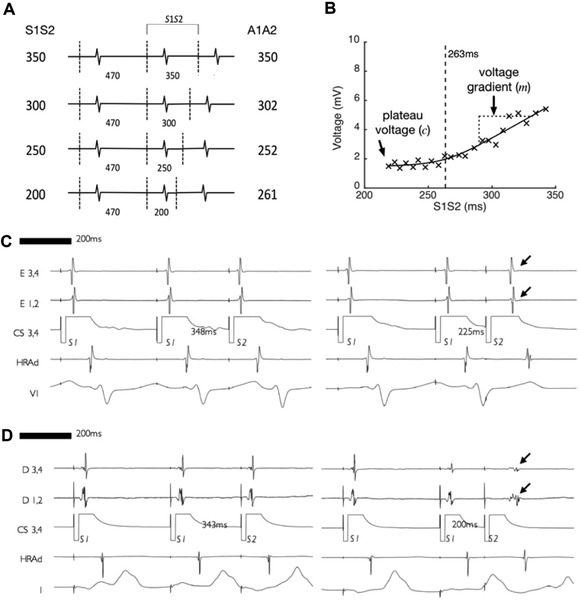
Pacing protocol overview and example electrograms. **A**: pacing protocol consisting of a two‐beat 470 milliseconds drive train followed by a single premature extrastimulus reducing in 2% steps from 350 to 200 milliseconds or loss of atrial capture. **B**: bipolar voltage of atrial electrograms recorded at a single site. Bipolar voltage is seen to attenuate as extrastimulus coupling interval is reduced. A best‐fit curve consisting of a hyperbola with asymptotes at y = c and y = mx is fitted to the data, allowing the plateau voltage (c) and the gradient of the initial phase of voltage attenuation (m) to be determined. **C**: An example of a recording site where bipolar voltage attenuation with extrastimulus coupling interval was minimal (arrowed). **D**: An example of a recording site where bipolar voltage attenuation with extrastimulus coupling interval was more pronounced (arrowed)

The pacing protocol was delivered using a custom‐built, institutionally approved stimulator and consisted of a two‐beat drive train (470 milliseconds) followed by a single premature extrastimulus. The S1S2 coupling interval was reduced continuously without operator interference in 2% steps from 350 to 200 milliseconds or loss of capture, e.g., 470‐470‐350, 470‐470‐343, etc. (Fig. 1A). All pacing stimuli were delivered at a voltage of at least twice threshold with a pulse width of 2 milliseconds. The PentaRay catheter was sequentially maneuvered to multiple sites in the body of the LA and bipolar electrograms were recorded throughout in response to complete S1S1S2 pacing trains delivered from the mid‐CS. Unipolar electrograms were recorded using the IVC electrode of the pentapole catheter as the indifferent electrode.

### 2.3 Signal processing

Electrograms were digitized using LabSystem Pro‐EP (Bard Electrophysiology) at 16‐bit / 4 kHz. Signal processing was performed offline (MATLAB 9.1, MathWorks, MA, USA). Pacing timing was determined from the paced channel. The first pacing cycle was used to determine the noise threshold and discarded from subsequent analysis. A2 electrograms were rejected from analysis where the signal was far‐field, there was fusion with an atrial ectopic beat or there was failure of capture of one or more of the two preceding S1 beats.

Bipolar voltage was defined as peak‐to‐peak electrogram voltage. To quantify the relationship between coupling interval and bipolar voltage, a hyperbola with asymptotes at y = c and y = mx was fitted to the data. From these curves the plateau voltage (*c*) and voltage gradient (*m*) were determined (Fig. 1B). For analysis of voltage at long and short extrastimulus coupling intervals, the point of intersection of the two asymptotes (e.g., 263 milliseconds for the recording site in Fig. 1B) was taken as the cut off to define long and short extrastimulus coupling intervals. Plateau cut off voltages of <0.5 mV and <1.0 mV are used to represent very low voltage and low voltage respectively, based on fifth centile voltages in a prior study of LA mapping using the PentaRay catheter.^21^

To allow processing of unipolar electrograms, a window of interest from the beginning to end of the corresponding bipolar signal was determined as follows. A band‐pass filter (30–500 Hz) was applied to bipolar recordings, and the noise threshold defined as signal mean ± 3SD of the 100 milliseconds preceding the S2 component of the first cycle. The first and last peaks exceeding the noise threshold were taken as the beginning and end of the bipolar signal, respectively. The unipolar voltage was then defined as the maximum peak‐to‐peak voltage of the unipolar signal within this time window. The unipolar activation time was defined as the steepest negative gradient (dV/dt) of the unipolar signal within the same time window.

### 2.4 Postablation follow‐up

Arrhythmia recurrence was documented over 2‐year routine clinical follow‐up consisting of 3‐ to 6‐monthly clinic review combined with 48‐hour ambulatory monitoring.

### 2.5 Statistical tests

Data analysis was performed using GraphPad Prism 7.0b (GraphPad Software, CA, USA). Data are represented as mean ± SD. Continuous variables were compared using Student's two‐tailed T‐test. Group means of three or more variables were compared with analysis of variance (ANOVA). Two‐way ANOVA was used to compare electrogram pattern frequencies between patients with and without arrhythmia recurrence. P < 0.05 was considered significant.

## 3 RESULTS

Ten patients (40% male, age 63 ± 12 years) undergoing first‐time pulmonary vein isolation were studied. All patients had PAF (duration of symptoms 2.9 ± 3.2 years) and similar left atrial dimensions (3.8 ± 0.4 cm). The median CHA_2_DS_2_Vasc score was 2 (interquartile range 0–4). There were no regions of LA low voltage (<0.3 mV, [11]) during baseline pacing. In total, electrograms were recorded at 119 ± 37 sites per LA. An operator blinded to the case details inspected the quality of every electrogram.

### 3.1 Bipolar electrograms

**Figure 2:**
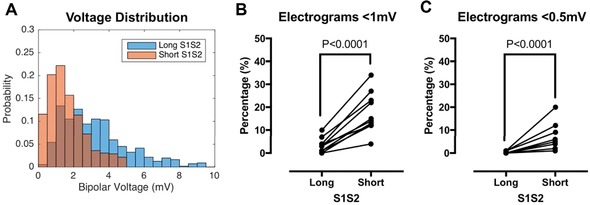
Effect of bipolar voltage attenuation on left atrial voltage quantification. **A**: Bipolar voltage distribution for all recording sites is shown at long extrastimulus coupling interval (blue bars) and short extrastimulus coupling interval (orange bars), with overlap between the bars shown in brown. **B**: Sites with voltage <1 mV (low voltage) were significantly more frequent at short compared to long extrastimulus coupling interval. **C**: Sites with voltage <0.5 mV (very low voltage) were significantly more frequent at short compared to long extrastimulus coupling interval. No sites showed very low voltage at long extrastimulus coupling interval [Color figure can be viewed at wileyonlinelibrary.com]

Example bipolar electrogram recordings from the beginning and end of the pacing train are given in Figures 1(C) and 1(D). In Figure 1(C), there is no attenuation of bipolar electrogram voltage at short compared to long extrastimulus coupling interval. In contrast, Figure 1(D) demonstrates a site where bipolar electrogram voltage attenuated as the extrastimulus coupling interval was shortened. Taking all recording sites together, a leftward skew of the bipolar voltage distribution histogram was evident when voltages were measured at short extrastimulus coupling interval compared to long extrastimulus coupling interval (Fig. 2A). Consequently, the proportion of electrograms showing low voltage (<1 mV) or very low voltage (<0.5 mV [21]) was significantly greater at short extrastimulus coupling interval compared to long extrastimulus coupling interval (Figs. 2B and C).

**Figure 3:**
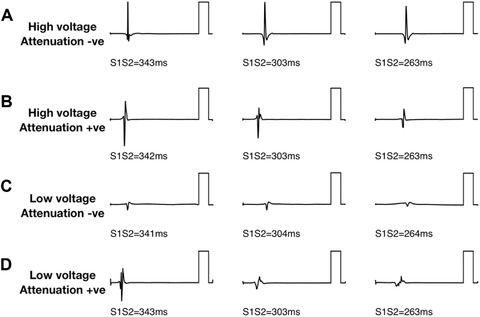
Bipolar electrogram voltage patterns. Four patterns of electrogram voltage response to extrastimulus coupling interval were identified. Example electrograms are presented for each pattern. **A**: high electrogram voltage regardless of coupling interval (“high voltage, attenuation –ve”). **B**: Attenuation of electrogram voltage with shortening extrastimulus coupling interval to not less than 1 mV (“high voltage, attenuation +ve”). **C**: low electrogram voltage irrespective of coupling interval (“low voltage, attenuation –ve”). **D**: Attenuation of electrogram voltage with shortening extrastimulus coupling interval to less than 1 mV (“low voltage, attenuation +ve”). Reference bars are 5 mV

**Figure 4:**
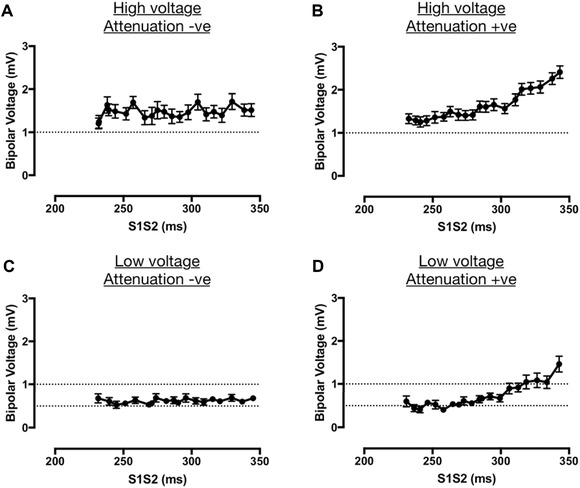
Bipolar electrogram voltage patterns. Four patterns of electrogram voltage response to extrastimulus coupling interval were identified. S1S2‐bipolar voltage curves are shown for all recording sites of each category. **A**: high electrogram voltage regardless of coupling interval (“high voltage, attenuation –ve”). **B**: Attenuation of electrogram voltage with shortening extrastimulus coupling interval to not less than 1 mV (“high voltage, attenuation +ve”). **C**: low electrogram voltage irrespective of coupling interval (“low voltage, attenuation –ve”). **D**: Attenuation of electrogram voltage with shortening extrastimulus coupling interval to less than 1 mV (“low voltage, attenuation +ve”)

Four patterns of bipolar electrogram voltage responses were distinguished according to the best fit voltage‐coupling interval curves. For each pattern, example electrograms are given in Figure 3 with mean voltage‐coupling interval curves given in Figure 4.

(1) “High plateau voltage/attenuation –ve” sites (HP; A–) were characterized by bipolar voltage >1 mV regardless of extrastimulus coupling interval, without voltage attenuation at long coupling intervals.
(2) “High plateau voltage/attenuation +ve” (HP; A+) sites were characterized by voltage attenuation at long coupling intervals with a plateau voltage >1 mV.
(3) Low voltage/attenuation negative sites (LP; A–) were characterized by low bipolar voltage (<1 mV) regardless of coupling interval, without voltage attenuation at long coupling intervals.
(4) “Low plateau voltage/attenuation +ve” sites (LP; A+) were characterized by voltage attenuation at long coupling intervals with a plateau voltage <1 mV.

**Figure 5:**
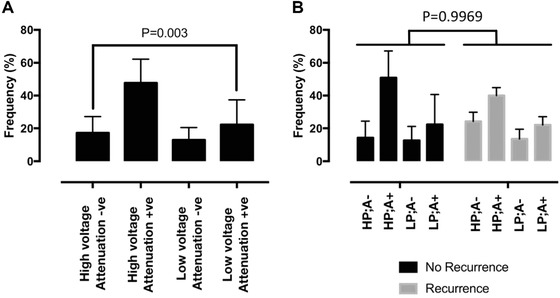
Frequency of occurrence of bipolar electrogram voltage patterns. Frequency of occurrences of each category of bipolar voltage patterns is shown. At both low and high voltage, attenuation +ve patterns were significantly more common than attenuation –ve patterns; with the high voltage, attenuation +ve pattern (HP;A+) being the most common pattern overall. **A**: frequencies for the entire study population. **B**: frequencies for patients with and without recurrence of arrhythmia postablation

The commonest pattern of electrogram voltage was HP; A+ (48 ± 15%), followed by LP; A+ (22 ± 15%), HP; A– (17 ± 10%) and LP; A– (13 ± 8%; Fig. 5A). There was no significant difference in these electrogram voltage patterns between patients with and without recurrence of arrhythmia post ablation (Fig. 5B), although this study was not powered to assess response to ablation.

### 3.2 Unipolar electrograms

**Figure 6:**
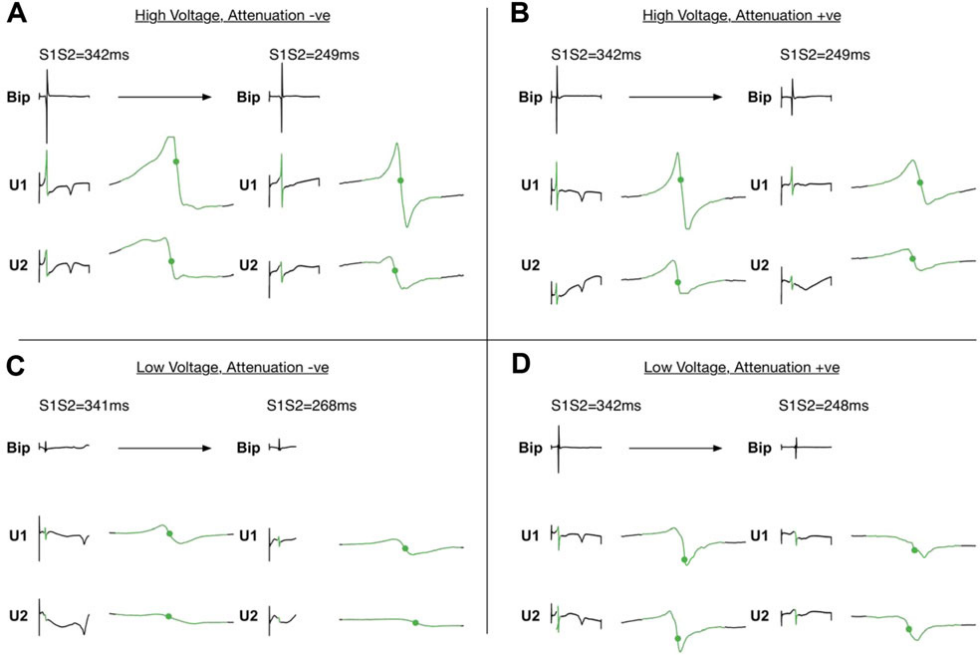
Unipolar electrogram patterns. Example unipolar electrograms recorded between the PentaRay and an IVC electrode are shown for each of the four identified bipolar voltage patterns. **A**: At high voltage, attenuation –ve sites there was minimal change in unipolar voltage and timing between long and short extrastimulus coupling interval. **B**: At high voltage, attenuation +ve sites corresponding attenuation of unipolar voltage and increase in unipolar delay (green dots) was seen. **C**: At low voltage, attenuation –ve sites unipolar voltage was again similar between long and short extrastimulus coupling interval. Note at this recording site there is also increased unipolar delay between U1 and U2 at short compared to long coupling interval. **D**: Low voltage, attenuation +ve sites were characterized by corresponding attenuation of unipolar electrogram voltage [Color figure can be viewed at wileyonlinelibrary.com]

**Figure 7:**
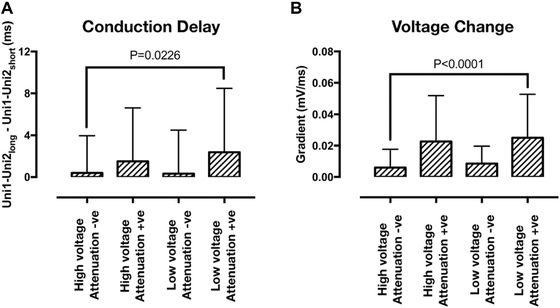
Unipole electrogram delay and unipolar voltage gradient. **A**: Quantification of conduction delay between unipolar pairs was performed by determining the difference in Uni1 and Uni2 activation times at short versus long extrastimulus coupling interval. There was a small but significant increase in conduction delay at sites showing extrastimulus coupling interval associated bipolar voltage attenuation. **B**: Quantification of unipolar voltage change with extrastimulus coupling interval was performed by determining the gradient of the initial voltage attenuation (m in Figure 1B) for unipolar signals. Unipolar voltage attenuation was significantly greater at sites demonstrating bipolar voltage attenuation

Figure 6 shows representative unipolar electrograms recorded at sites showing each of the four bipolar electrogram voltage patterns. Sites showing bipolar electrogram voltage attenuation also generally showed unipolar voltage attenuation (Figs. 7B and D). In contrast, unipolar electrograms recorded at sites with no attenuation of bipolar electrogram voltage were similar at both long and short extrastimulus coupling interval (Figs. 7A and C). On a site‐by‐site basis, the relationship between bipolar electrogram voltage and conduction delay between unipolar pairs was less clear. Unipolar activation times are annotated with green dots in Figure 6. Some sites showed changes in U1‐U2 activation time difference as extrastimulus coupling interval was reduced (Fig. 6B), whereas at other sites, the U1‐U2 relationship was fixed regardless of coupling interval (Fig. 6A). Quantification of these observations is given in Figure 7 indicating that, overall, sites showing bipolar voltage attenuation (both high plateau voltage and low plateau voltage) were associated with significantly greater change in unipolar voltage at long coupling intervals (Fig. 7B) and significantly greater conduction delay between unipole electrodes at short coupling intervals.

## 4 DISCUSSION

In this study, extrastimulus pacing from the coronary sinus was used to determine the relationship between bipolar electrogram voltage and extrastimulus coupling interval. The main findings of the study are as follows: (1) Four patterns of bipolar voltage / extrastimulus coupling interval curves are identifiable, characterized by either high or low plateau voltage at short coupling interval and either high or low initial voltage attenuation gradients at long coupling intervals. (2) High plateau voltage, with an initial voltage attenuation gradient, is the pattern most frequently encountered, followed by low plateau voltage with an initial attenuation gradient. Sites without a voltage attenuation gradient, with either high or low plateau voltages are less frequently encountered. (3) Changes in bipolar voltage associated with shortening extrastimulus coupling interval are mirrored by changes in unipolar voltages recorded at the same site, although there is also a modest but significant increase in unipole activation times indicative of local conduction delay at sites showing an initial voltage attenuation gradient at long coupling intervals.

In recent years there has been a growing body of literature focusing on the role of voltage mapping to define the atrial arrhythmia substrate, based on the premise that areas of abnormal tissue will be characterized by low bipolar voltage. Whilst regions of very low or undetectable bipolar voltage are likely to indicate abnormal regions of atrial myocardium,^1^ the significance of low bipolar voltage above the low voltage threshold, and its relationship to components of the arrhythmia substrate has not been conclusively demonstrated. Indeed the association between low voltage and CMR indices of fibrosis (which provide the only current noninvasive atrial tissue characterization method) is relatively weak, with significant overlap between areas of enhancement and both low and high voltage regions. Based on the findings of the present study, it seems reasonable to conclude that a more sophisticated analysis of bipolar voltage may be necessary to detect atrial fibrosis using electroanatomic mapping, and that use of a simple static voltage threshold may contribute to the limited correlation between fibrosis and voltage in some studies.^9^, ^10^

### 4.1 Voltage mapping to define ablation targets

A number of observational studies have recently used voltage mapping to define ablation targets. Cutler et al. used the presence of LA voltage <0.5 mV in the posterior LA to select patients for posterior LA box isolation, and reported improved outcomes compared to standard ablation.^7^ Similarly, Kottkamp et al. identified low voltage areas (<0.5 mV) in 60% of nonparoxysmal AF patients and found that box isolation of these areas could successfully treat AF in these cases.^6^ In both of these studies voltage mapping was performed during sinus rhythm. Since the sinus rate was not documented it is not possible to determine whether atria activating with a higher sinus rate were more likely to demonstrate low voltage areas. Given the results of the present study, however, it would appear essential to consider the sinus activation rate when selecting voltage‐guided ablation strategies or, as is our preferred approach, to perform voltage mapping under CS pacing at the same cycle length in every patient.

Numerous other studies have also assessed the role of substrate‐guided ablation for paroxysmal and persistent AF, and have been able to demonstrate significantly improved outcomes compared to anatomical based ablation (i.e., PVI or PVI plus linear ablation). These studies include strategies based on electrogram voltage alone^5^, ^22^, ^23^ or electrogram voltage interpreted in the context of other electrogram morphological criteria.^24^ Notably however, despite improved outcomes, the recurrence rates for atrial arrhythmias in these studies is still around 20% at 1‐year follow‐up. Based on the present study, part of the reason for these still‐limited success rates may be inadequate identification of true low voltage/substrate regions owing to failure to map at rapid activation rates. Whether such dynamic voltage mapping and targeted ablation can lead to further improvements in outcomes in persistent AF ablation should be assessed in future studies.

### 4.2 Mechanisms underlying voltage attenuation patterns

A number of physical factors can affect bipolar voltage recordings. For example, electrode size and spacing are important determinants of bipolar voltage recorded in the atrium.^21^ Since the bipolar signal is created by amplifying the potential difference between two closely spaced recording electrodes, differences in activation timing at each electrode could also affect bipolar voltage. In order to determine whether the changes in bipolar voltage observed here arose due to changes in unipolar activation times, or whether changes in bipolar voltage were underpinned by changes in unipolar voltage, unipolar electrograms were recorded between the PentaRay catheter and an indifferent electrode in the IVC. We found that sites showing bipolar voltage attenuation at long coupling intervals also showed unipolar voltage attenuation at the same coupling intervals, indicating that changes in unipolar voltage are at least associated with the changes identified in bipolar voltages. Analysis of the unipolar recordings also found a small but significant increase in conduction delay at sites showing bipolar voltage attenuation. In this regard, it is interesting to note that sinus rhythm voltage is reduced in the presence of atrial fibrosis in areas with reduced connexin (Cx40 and Cx43) expression in heart failure dogs,^25^ indicating a likely interplay between conduction and voltage in this model. One hypothesis arising from these data is that fixed reductions in voltage (i.e., A and B vs. C and D in Fig. 4) depend more on reduced gap junction density, potentially arising due to fibroblast proliferation, whereas rate‐dependent effects on voltage (i.e., B and D vs. A and C in Fig. 4) depend more on dynamic changes in sodium channel function.^26^ Further *ex vivo* studies will be required to determine the full extent of the relationship between these phenomena and their association with atrial fibrosis.

The effect of atrial wall thickness on static and dynamic voltage patterns should also be considered. The posterior atrial wall is significantly thinner than other parts of the atria, and correspondingly, during mapping with a 3.5‐mm distal tip ablation catheter, Kapa et al. demonstrated that voltage cut‐offs in the posterior wall and LA‐PV junctions are significantly lower than elsewhere in the atria (<0.2 mV vs. <0.45 mV).^27^ It is reasonable to speculate that regions of thicker atrial myocardium, or areas of wall thickness heterogeneity, may influence the rate‐dependence of electrogram voltage owing to differences in endocardial–epicardial activation at the mapping site,^28^ or alterations in local conduction.^29^

### 4.3 Clinical implications

Despite advances in ablation technologies, the success rates for AF ablation, and in particular persistent AF ablation, remain modest. As discussed above several recent studies have shown success in targeting low voltage areas (presumed atrial fibrosis) in improving outcomes for both paroxysmal and persistent AF ablation. Nevertheless, there are still patients in whom these strategies fail to restore sinus rhythm. Part of the reason for these limitations may be the relatively low sensitivity with which bipolar voltage mapping (performed at a single activation rate) can identify fibrotic atrial myocardium, as shown by the weak correlation between bipolar voltage and CMR indices of atrial fibrosis.^8^, ^9^ The data presented here indicate that bipolar voltage, at a majority of sites, attenuates significantly as activation rate is increased. Furthermore, through analysis of corresponding unipolar signals we show that these changes are explained both in terms of unipolar amplitude and activation delay. Overall, these findings illustrate that dynamic voltage mapping (taking into account activation rate) can uncover significant electrophysiological heterogeneity between patients with paroxysmal AF, and in doing so may improve the sensitivity for detection of fibrotic myocardium. Potential clinical implications of these observations are as follows:

(1) First, standard criteria for voltage mapping should be developed. As a starting point we would suggest that LA voltage mapping should be performed under pacing conditions rather than sinus rhythm in order to create uniformity between all patients studied. CS pacing at 500–600 milliseconds cycle length would seem a reasonable approach to avoid significant voltage attenuation and potential overestimation of atrial scar.
(2) Second, the clinical utility of voltage mapping at shorter cycle lengths, or voltage mapping at multiple cycle lengths should be determined. For example, dynamic voltage mapping may indicate patients with more extensive atrial disease who are less likely to respond to index, or repeat ablation. Alternatively, and arguably more hypothetically, rate dependence of bipolar voltage may be able to better define targets for radiofrequency ablation. Both hypotheses should be tested in future studies.

### 4.4 Limitations

The effect of activation rate on bipolar voltage was analyzed in this study only for LA activation arising from a single pacing site (mid CS). The mid CS was chosen to ensure that the LA activated uniformly. Previous studies have shown discrepancies between LA voltage under HRA and CS pacing conditions,^30^ potentially due to fusion of LA activation via the septum and Bachman's bundle. Whether different voltage‐rate effects would be revealed by alternative activation vectors is not answered by these data but is worthy of further study, particularly given the lack of relationship between LA voltage in sinus rhythm and AF (where multiple, chaotic activation vectors are likely to be present).^31^

Voltage recordings were made using the PentaRay catheter. Since this is a non‐force sensing catheter it is not possible to confirm uniform catheter‐myocardium contact force across all sites studied. Nevertheless, in a recent analysis using CartoSound, Sasaki et al. showed no significant relationship between voltage and contact force,^32^ indicating that contact force is unlikely to have had a significant effect on the voltage data presented here. A further limitation may be incomplete coverage of the atrium using the PentaRay catheter. The mean mapping density was 0.45 ± 0.15 points/cm^2^, which was limited by the long pacing train required to collect all the S2 extrastimulus electrograms. Nevertheless, performing detailed extrastimulus mapping allowed the complexity of the relationship between activation rate and voltage to be determined. Future studies, using abbreviated versions of the protocol, would be required to delineate the spatial distribution of these voltage patterns.

In this study, we tested the hypothesis that electrogram voltage is activation rate dependent. By examining a group of patients with apparently healthy atria (i.e., no low voltage by standard criteria) we showed that rapid pacing can reveal differences in electrophysiology between patients, through revealing areas of voltage attenuation in some but not other patients. It is likely that different distributions of these voltage patterns would be seen in patients with more frequent, or persistent, AF.

## 5 CONCLUSION

In conclusion, bipolar electrogram voltage is significantly influenced by activation rate at a majority of LA recording sites in patients with paroxysmal AF. Activation rate should therefore be consistent when comparing voltage measurements made both within and between patients. Studies using bipolar voltage to define an arrhythmia substrate should specify the pacing or sinus rates at which voltage maps were created. Further research is necessary to define the relationship between these bipolar voltage patterns and individual components of the arrhythmia substrate.
